# Synaptic micromechanics and brain softening as a mechanobiological hypothesis for Alzheimer’s disease

**DOI:** 10.3389/fnins.2026.1858655

**Published:** 2026-06-17

**Authors:** Denis Le Bihan

**Affiliations:** NeuroSpin, Joliot Institute, CEA, CEA-Saclay Center, Paris-Saclay University, Gif-sur-Yvette, France

**Keywords:** Alzheimer’s disease, brain stiffness, diffusion MRI, gamma stimulation, glymphatic system, magnetic resonance elastography, mechanobiology, neuromechanical coupling

## Abstract

Alzheimer’s disease (AD) is usually framed as a proteinopathy and network disorder, but this view may be incomplete. We propose a mechanobiological hypothesis in which synaptic micromechanics, regional brain softening, vascular pulsatility, and glymphatic transport are parts of a coupled fluid–solid system whose failure contributes to AD progression. In this framework, early synaptic and glial mechanical fragility reduces the capacity of vulnerable circuits to maintain stable structure, efficient signaling, and waste clearance, while age-related tissue softening and impaired perivascular transport amplify amyloid and tau accumulation, network dysfunction, and cognitive decline. This framework integrates converging evidence from dendritic spine to glymphatic system biology, concordant results obtained with diffusion MRI and magnetic resonance elastography, and treats altered tissue mechanics not merely as a correlate of degeneration but as a potentially active multicomponent of disease expression. It further predicts that biomechanical alterations should be detectable before gross atrophy, should covary with glymphatic impairment, and may help explain why molecular pathology and clinical symptoms are often only partly aligned. By positioning brain mechanics as an interface between protein aggregation, synaptic dysfunction, and impaired clearance, this framework identifies testable imaging biomarkers and suggests potential early-stage intervention strategies aimed at preserving tissue resilience as well as reducing pathological protein burden.

## Introduction

Brain function has traditionally been interpreted through two tightly linked frameworks: neurochemistry and electrophysiology. The modern history of the synapse begins with Santiago Ramón y Cajal (1911), who argued that the nervous system is composed of discrete neurons that come into close apposition without forming a continuous network. Charles Sherrington then named this junction in 1897, proposing the term “synapsis,” later standardized as “synapse” (Sherrington, 1897). In 1921, Otto Loewi provided decisive experimental evidence that transmission across this junction could be chemical (Loewi, 1921), and Dale et al. (1936) work on acetylcholine helped generalize that finding into the broader concept of neurotransmission. In parallel, electrophysiology supplied the biophysical language of neural signaling. This line of work culminated in the classic studies of Hodgkin and Huxley (1952), which showed that the action potential arises from voltage- and time-dependent ionic conductances across the membrane (Hodgkin et al., 1952; Hodgkin and Huxley, 1952). Together with later work on synaptic potentials and spike propagation (Eccles et al., 1954; Coombs et al., 1955), these studies established membrane polarization, synaptic integration, and impulse propagation as the canonical architecture of neural communication.

**Figure 1:**
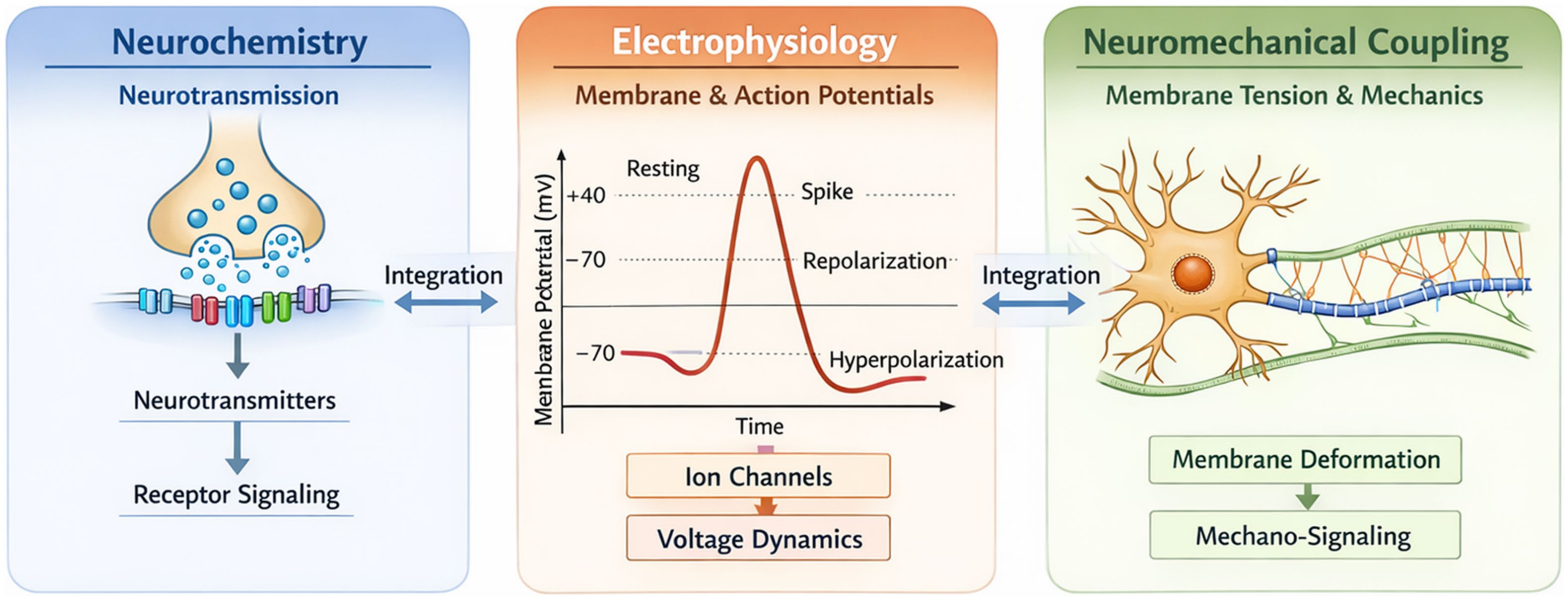
Integration of neurotransmission, electrophysiology and neuromechanical coupling.

This framework has been extraordinarily successful, shaping modern neuroscience from cellular physiology to psychopharmacology (Albright et al., 2000). At the same time, its historical dominance helps explain why alternative dimensions of brain function, such as metabolic, mechanical, glial, or electromagnetic influences (Barnes et al., 2017), were long treated as secondary to the electrochemical model rather than as potentially coequal components of neural signaling. Yet the standard formulation leaves one important conceptual space only partially developed: many of the processes it describes are also mechanical. Synapses are membrane-bound structures whose geometry and compliance influence transmission and plasticity (O’Donnell et al., 2011; Araya et al., 2014); neurons and glia undergo activity-dependent deformation; the extracellular space is a deformable transport medium; vascular pulsatility shapes perivascular flow; and regional tissue stiffness changes with ageing and neurodegeneration (Pavuluri et al., 2025) (Figure 1).

Importantly, this mechanobiological perspective should not be understood as a single linear mechanism operating uniformly across levels of organization. Rather, it is a multiscale framework that encompasses distinct but potentially convergent mechanical processes, from dynamic membrane and cytoskeletal deformation at the level of dendritic spines to extracellular-space remodeling, vascular pulsatility, glymphatic transport, and regional changes in brain shear stiffness. These elementary mechanisms are not necessarily causally reducible to one another. For example, impaired spine micromechanics may alter synaptic signaling and plasticity without directly causing macroscopic tissue softening, whereas reduced parenchymal stiffness may reflect demyelination, extracellular-matrix remodeling, vascular ageing, altered fluid content, or tissue rarefaction. The strength of this view is therefore not that it proposes a single mechanical lesion, but that it provides a common conceptual framework for otherwise distinct processes through which brain tissue may lose mechanical resilience across spatial scales.

A mechanobiological framework for Alzheimer’s disease (AD) could therefore extend our current understanding of the disease as a whole, helping distinguish molecular lesion burden from mechanical operating range. Alzheimer’s disease (AD) is now understood as a progressive biological cascade in which abnormal amyloid-β handling, tau misfolding and spread, synaptic failure, glial activation, vascular/metabolic stress, and network disconnection converge over years before dementia becomes clinically obvious (Zhang et al., 2024). Brain changes usually begin long before symptoms; amyloid plaques accumulate extracellularly, tau pathology first appears in memory-related medial temporal structures and then spreads more widely, and neurons gradually lose their ability to communicate, survive, and support cognition (Montoliu-Gaya et al., 2025). This model has been highly productive. It explains why amyloid biomarkers become abnormal years before symptoms, why tau burden tracks neuronal injury and cognitive decline more closely than amyloid alone, and why the earliest clinical manifestations involve memory circuits centered on the entorhinal cortex and hippocampus. It also underlies the current shift toward biomarker-guided treatment in early symptomatic disease (Cummings et al., 2025; Zhang et al., 2024).

Still, proteinopathy, synaptic failure, fluid transport, and tissue mechanics may be coupled across scales. One key mechanism linking pathology to symptoms is synaptic dysfunction. Two synapses may experience similar amyloid exposure yet differ in their ability to maintain spine geometry, membrane trafficking, and local ionic balance under repeated activation. The synapse with lower mechanical reserve may fail first, thereby destabilizing the surrounding circuit. This perspective could help explain why symptom emergence is often nonlinear and why network dysfunction can appear disproportionate to overt histopathological load in early disease. Long before extensive neuronal death or overt atrophy, soluble amyloid species and pathological tau appear to impair synaptic transmission, plasticity, and circuit stability (Zhang et al., 2024). Clinically, this matters because synapse loss correlates strongly with cognitive decline. Memory impairment, slowed thinking, loss of mental flexibility, and reduced capacity to manage multistep tasks can all be understood as manifestations of progressive failure in distributed synaptic networks rather than simply as “cell death” in isolated regions (Zhang et al., 2024).

An additional mechanism that has attracted growing attention is failure of brain protein and fluid clearance through the glymphatic system, a brain-wide perivascular transport and clearance pathway in which cerebrospinal fluid enters along periarterial spaces, exchanges with interstitial fluid within the parenchyma, and exits along perivenous and meningeal lymphatic routes. AD relevance is biologically plausible because impaired glymphatic/meningeal lymphatic clearance—exacerbated by ageing, sleep disruption, vascular stiffening and AQP4 depolarization—may promote amyloid-β, tau and inflammatory mediator accumulation (Ding et al., 2025). Although this remains an active area of research rather than settled clinical doctrine, impaired clearance of amyloid, tau, and inflammatory mediators is increasingly considered a plausible contributor to disease progression. This is especially relevant because sleep disruption, vascular stiffness, and aging may all worsen clearance efficiency, potentially helping explain why sleep fragmentation and circadian disturbance are both common symptoms and possible disease accelerants (Zhang et al., 2024).

The value of such a multiscale mechanobiological framework is threefold. First, it may help explain why symptoms can emerge while tissue still appears relatively preserved on conventional structural MRI: a mechanically fragile, but not yet grossly atrophic, network may already be functionally compromised. Second, it is compatible with the concept of selective vulnerability. Regions such as the entorhinal cortex and hippocampus rely on exceptionally elaborate dendritic and spine architectures. Tissue composed of densely ramified, mechanically dynamic compartments may be especially efficient for memory processing, yet especially vulnerable to chronic disturbances in cytoskeletal regulation, osmotic balance, and extracellular homeostasis. The very features that support mnemonic flexibility may therefore also confer mechanical fragility under proteinopathic stress (O’Donnell et al., 2011; Araya et al., 2014). Third, it may identify therapeutic opportunities, especially in early disease, considering the failure of protein and fluid clearance through dysfunction of the glymphatic system. That are missed by a purely protein-centric view.

In summary, the purpose of this article is not to claim that altered tissue mechanics are the primary cause of AD in any simple upstream sense. Rather, the aim is to articulate a framework in which molecular pathology, synaptic dysfunction, vascular ageing, clearance failure, and regional softening are interpreted as mutually reinforcing components of a single degenerative system. In this view, the disease begins in vulnerable synaptic microdomains, progresses through the neuronal–glial–extracellular matrix that supports local computation, and becomes visible at the regional level as altered viscoelasticity, impaired pulsatile transport, and declining network resilience. This formulation remains hypothesis-generating, but it may yield specific predictions about biomarkers, disease staging, and early intervention.

## Synaptic micromechanics and early vulnerability

Synaptic dysfunction is among the earliest and strongest correlates of cognitive decline in AD. Soluble amyloid species, tau-mediated cytoskeletal disruption, calcium dysregulation, mitochondrial stress and inflammatory signaling all impair synaptic transmission and plasticity before widespread neuronal loss occurs. This is especially important in medial temporal memory networks, where episodic memory formation depends on dense assemblies of highly plastic excitatory synapses. Most of these excitatory synapses are located on dendritic spines.

### Neuromechanical coupling

**Figure 2:**
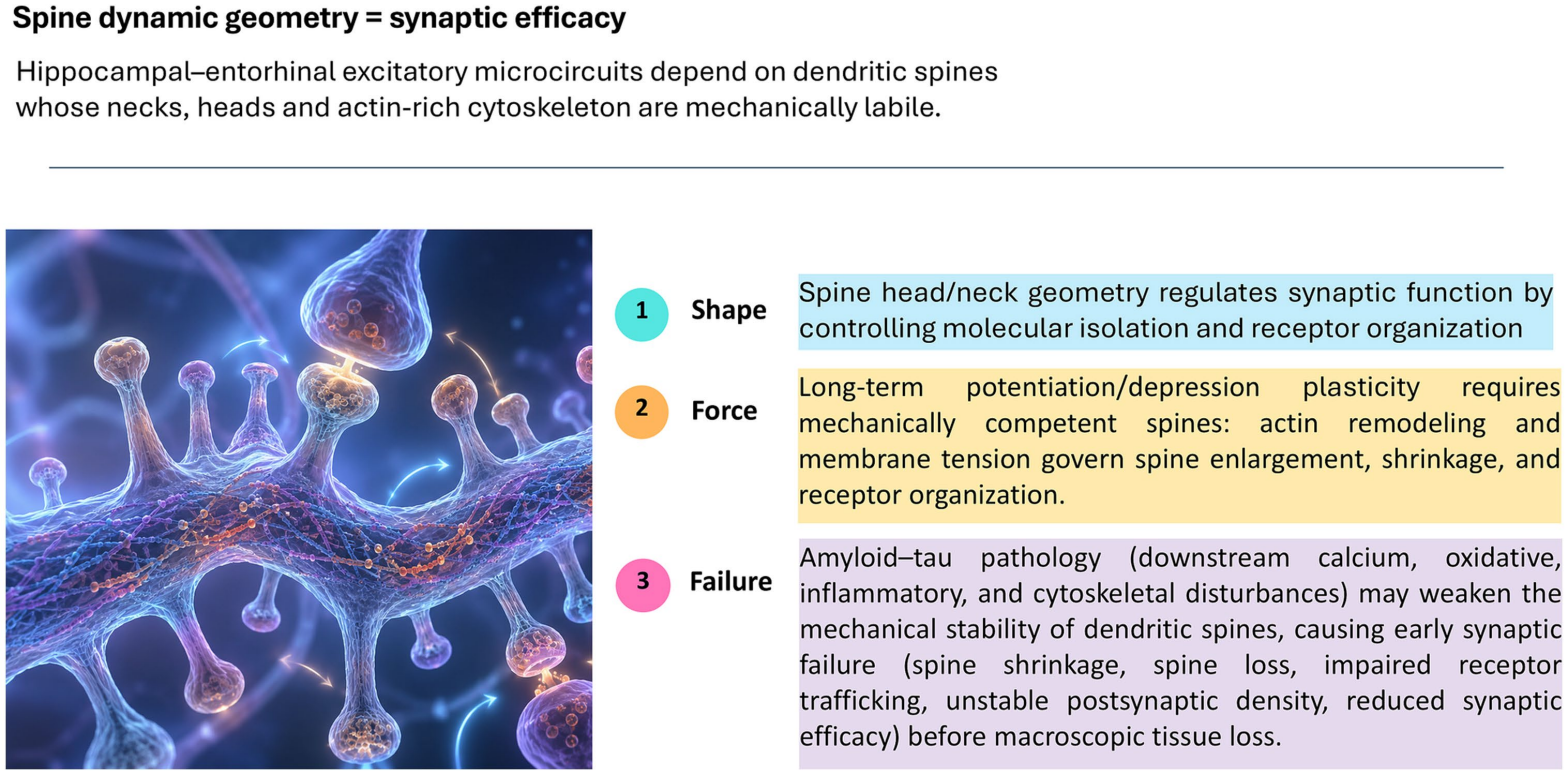
Spine dynamic geometry features and their failure link in relation to amyloid-tau pathology.

The role of dendritic spines deserves particular emphasis because they offer the clearest concrete substrate for linking neural activity to mechanics. Spines are actin-rich protrusions with high membrane curvature, a narrow neck and a bulbous head that contains the postsynaptic density. Their geometry determines electrical filtering, calcium compartmentalization and molecular diffusion between the spine and the dendritic shaft. Hence, dendritic spines are not passive appendages, as already envisaged long ago by Crick (Crick, 1982). They are mechanically and biochemically specialized microcompartments whose size, neck geometry, membrane tension and actin organization influence synaptic efficacy (Figure 2) (O’Donnell et al., 2011; Araya et al., 2014). Early studies have identified that neural swelling upon activation involves a dense polymer-gel matrix of cross-linked actin filaments and microtubules running contiguously to the cell membrane (Metuzals and Tasaki, 1978; Endo et al., 1979). It has been shown that agents which disrupt microtubules or solubilize gels, and thus eliminate the cytoskeleton, suppress the action potential (Tsukita et al., 1986; Matsumoto et al., 1979). Cytoskeletal integrity, thus, appears necessary for the action potential to occur (Metuzals and Tasaki, 1978). More recently, atomic force microscopy (AFM) measurements on living dendritic spines revealed viscoelastic, soft-glassy behavior (Smith et al., 2007), and subsequent work placed actin polymerization, membrane deformation, and clutch-like coupling between the actin cytoskeleton and adhesion complexes at the center of synapse formation and plasticity (Kastian et al., 2021; Minegishi et al., 2023); this mechanical perspective is important because spine head size correlates with postsynaptic density and AMPA (*α*-amino-3-hydroxy-5-methyl-4-isoxazolepropionic acid) receptor content (Arellano et al., 2007; Matsuzaki et al., 2001), whereas spine neck geometry regulates electrical resistance, calcium compartmentalization, and biochemical exchange with the parent dendrite (Bloodgood and Sabatini, 2005; Grunditz et al., 2008; Hugel et al., 2009). Activity-dependent enlargement of the spine head accompanies potentiation and stabilizes strengthened synapses, whereas changes in neck length or width modulate the biochemical isolation of the compartment. These are not subtle epiphenomena but core elements of synaptic plasticity (O’Donnell et al., 2011; Araya et al., 2014).

The morphology of the spine is inseparable from the function of the synapse. Although neurons are not classical piezoelectric sensors, they do exhibit electromechanical coupling that resembles piezoelectric behavior in some respects. Membrane-potential changes can produce mechanical deformation, and mechanical stress can in turn influence electrical state. This coupling has been demonstrated in multiple ways. Mechanical waves have been linked to action-potential propagation (Cohen et al., 1968; Iwasa and Tasaki, 1980; Tasaki and Iwasa, 1982; Tasaki and Iwasa, 1982). The deformation is synchronous with the electrical signal and is not simply an osmotic phenomenon; rather, it is consistent with an electromechanical surface effect. The cell membrane can therefore be viewed as an electrostrictive capacitor in which voltage-dependent electric fields alter surface tension, while mechanosensitive channels such as Piezo1, Piezo2, TREK-1, and TRPV4 provide routes through which deformation can influence ionic currents.

### Implications for Alzheimer’s disease

From a physical standpoint, each activated synapse is also a site of osmotic and ionic stress. Fast components of neuromechanical coupling may arise from electromechanical membrane stress, because changes in transmembrane voltage can alter surface charge and membrane tension almost instantaneously (Iwasa et al., 1980). Under physiological conditions these shifts are tightly regulated and reversible, but under stronger challenge somata and dendrites can swell, dendrites can bead, and spines can disappear and later re-emerge at their original synaptic sites if the insult is brief (Hasbani et al., 2001). Neuromechanical coupling can therefore be viewed as the low-amplitude physiological end of a continuum whose pathological extreme is overt swelling, beading and structural failure (O’Donnell et al., 2011; Araya et al., 2014). These properties tie spine mechanics directly to memory: long-term potentiation is accompanied by spine enlargement and structural stabilization, whereas long-term depression and pathological stress drive shrinkage, neck remodeling, beading or spine loss (Figure 2) (O’Donnell et al., 2011). During potentiation, F-actin assembly enlarges and stabilizes the spine head, and experience-induced spines can remain stable for prolonged periods (Yang et al., 2009). In ageing human temporal cortex, larger spine heads predict better episodic memory performance even after accounting for classical Alzheimer pathology (Walker et al., 2024). Memory therefore depends not only on molecular signaling but also on the ability of spines to enter and maintain mechanically stable structural states.

This mechanobiological framework suggests that early AD may be understood partly as a failure of synaptic mechanocompetence. In healthy tissue, synapses repeatedly convert electrical and biochemical events into transient structural adjustments (O’Donnell et al., 2011). At the spine head and neck, geometry regulates local resistance, biochemical compartmentalization and exchange with the parent dendrite (Araya et al., 2014). If amyloid and tau disturb these shape-dependent operations, synaptic dysfunction is not simply a matter of altered transmitter release or receptor number; it is also a matter of altered physical behavior within synaptic microstructures. In AD, many of the biochemical insults known to impair synapses also act on volume-regulatory and cytoskeletal pathways. Amyloid oligomers perturb calcium homeostasis, tau disrupts transport and cytoskeletal organization, inflammatory mediators alter membrane trafficking, and mitochondrial dysfunction compromises the energy supply required for ionic pumping (Figure 2). Under these stresses, a spine or dendrite may remain anatomically present yet mechanically unstable or insufficiently adaptable, so may fail to support normal computation, plasticity or memory encoding (O’Donnell et al., 2011; Araya et al., 2014). In this view, early synaptic failure may arise from a progressive mismatch between electrical demand and mechanical adaptability across spine-rich networks.

This line of thinking is consistent with the broader observation that in AD, cognitive impairment correlates more closely with synapse loss and network dysfunction than with plaque burden alone (John and Reddy, 2020; Taddei and Duff, 2025). Plaques may initiate a vulnerable biochemical environment, but the clinical syndrome is more likely to arise from failure of distributed synaptic systems. The spine-rich dendritic arbor is therefore a plausible substrate for early disease-relevant mechanical abnormalities.

The same is true of perisynaptic astrocytic processes, which contribute to glutamate and potassium clearance, volume regulation and water transport (Cheung et al., 2015; Zhou et al., 2022; Walch and Fiacco, 2022). Neuronal spine mechanics and astrocytic mechanobiology are closely linked through the tripartite synapse. Astrocytic processes surround many synapses and dynamically regulate extracellular space, glutamate and ion buffering, water movement, metabolic support, and inflammatory signaling. Because astrocytes are mechanically sensitive cells, changes in spine activity and synaptic geometry can alter local astrocytic morphology, Ca^2+^ signaling, extracellular-space volume, and gliovascular coupling. Conversely, astrocytic stiffness, process motility, extracellular matrix remodeling, and AQP4-dependent water handling can influence synaptic shape, receptor trafficking, and the mechanical environment in which spines operate. Thus, spine mechanics and astrocytic mechanobiology form a coupled neuron–glia mechanical system linking synaptic plasticity, tissue viscoelasticity, and perivascular fluid transport (see below). If neuronal and glial microdomains jointly shape local deformability and diffusion restriction, then AD-related injury to either compartment could destabilize the synaptic mechanical environment. In that sense, early disease may already involve local fluid–solid uncoupling at the scale of tripartite synapses.

A further implication is that the traditional distinction between ‘functional’ and ‘structural’ pathology becomes less sharp. Synaptic plasticity is itself structural at the nano- to microscale. If a brain region is repeatedly challenged by impaired ion handling, inflammatory cytokines, tau-related transport failure and altered extracellular chemistry, its synapses may lose not only molecular fidelity but also the capacity for the small, rapid morphological adjustments that support physiological signaling (Pekny et al., 2016; Hall et al., 2021). There is not yet a definitive *in vivo* biomarker for this class of dysfunction, but diffusion-based methods and biomechanics-sensitive imaging, including magnetic resonance elastography, may offer routes toward detecting it (see below) (Kamagata et al., 2021; Hall et al., 2021). In this framework, synaptic failure is a defect of dynamic architecture as much as of molecular composition.

In Alzheimer’s disease, this mechano-structural system is perturbed across scales. Soluble Aβ oligomers can reduce neuronal membrane stiffness (Ungureanu et al., 2016), brain tissue often softens globally and regionally even while plaque-associated microenvironments may become locally stiffer (Murphy et al., 2016; Hu et al., 2023), and actin- and tau-related synaptic pathology undermines the machinery that stabilizes spine shape, receptor trafficking and clustered synaptic organisation (Pelucchi et al., 2020; Mijalkov et al., 2021). Together, these observations suggest that Alzheimer disease weakens the physical substrate of memory before extensive neuronal loss becomes obvious.

A related advantage of a mechanical perspective is that it makes compensation more concrete. Brains can tolerate substantial amyloid pathology before symptoms emerge. One explanation is cognitive reserve; another, not incompatible with it, is mechanical reserve. Networks may continue to function because synapses, glia and extracellular space remain sufficiently adaptable to preserve signaling despite molecular stress (O’Donnell et al., 2011; Araya et al., 2014). Clinical decompensation would then occur when this reserve is exhausted, allowing small molecular insults to produce large functional failures. Although speculative, this hypothesis offers a tangible tissue-level interpretation of reserve.

This framework also invites a reinterpretation of neuroinflammation. Microglia and astrocytes are often discussed as immune actors, yet they are also structural cells that remodel processes, occupy extracellular space and regulate water handling (Pekny et al., 2016; Sriram et al., 2024). Reactive gliosis can therefore alter the mechanical and diffusional microenvironment before overt neuronal death (Pekny et al., 2016). In some contexts these glial responses may be protective, temporarily stabilizing tissue architecture or facilitating debris clearance; in others they may stiffen local barriers or distort perisynaptic geometry (Pekny et al., 2016; Hall et al., 2021). Distinguishing these roles will be essential for integrating glial biology into an AD mechanobiology.

This mechanistic emphasis also sharpens the therapeutic question. If synaptic dysfunction includes impaired local shape dynamics, then interventions that preserve actin stability, calcium buffering, membrane trafficking, astrocytic water handling or extracellular ionic homeostasis may have disease-modifying relevance beyond their purely biochemical effects (John and Reddy, 2020; Zhou et al., 2022). Although such approaches remain largely preclinical, they fit naturally within a framework that places micromechanics near the origin of cognitive decline rather than treating it as a secondary manifestation of later degeneration (Hall et al., 2021).

## Diffusion MRI, tissue microstructure, and neuromechanical coupling

### Diffusion MRI and tissue microstructure

Over the last 40 years diffusion MRI has had a transformative impact on medical imaging, particularly used to investigate and manage many neurological disorders, brain connectomics, neurodegenerative and psychiatric disorders (Le Bihan, 2024). Diffusion MRI allows brain tissue microstructure to be investigated noninvasively *in vivo* through the Brownian motion of water molecules is sensitive to cellular and subcellular barriers. Over diffusion times used in MRI (50-80 ms), water typically diffuses only around a few micrometers, so its displacement is shaped by membranes, myelin, axons, dendrites and extracellular geometry (Le Bihan, 2003). MRI encodes these microscopic displacements into measurable signal attenuation, thereby linking molecular motion to tissue architecture. In brain tissue it becomes hindered, restricted and compartmentalized, meaning that the signal reflects an ensemble average of intracellular and extracellular environments. In white matter, where aligned axons impose directional constraints on water movement, water diffuses more easily along fiber bundles than across them, producing anisotropy that can be captured through the Diffusion Tensor Imaging (DTI) framework (Basser et al., 1994). From the tensor, metrics such as mean diffusivity and fractional anisotropy provide quantitative, though indirect, markers of tissue integrity and organization, while tractography extends these local orientations into estimates of large-scale structural connectivity (Le Bihan, 2003). More advanced approaches such as diffusion kurtosis imaging) (Jensen et al., 2005) and NODDI (neurite orientation dispersion and density imaging (Zhang et al., 2012) exploit non-Gaussian diffusion to improve sensitivity to tissue complexity, neurite density and orientation dispersion. Overall, diffusion MRI enables noninvasive interrogation of cellular and subcellular organization that are otherwise available only through local microscopy, repeatedly, without contrast agents or ionizing radiation, and over the entire brain. As a result, diffusion MRI acts as a virtual biopsy, offering noninvasive access to the organization, density, and integrity of neural tissue, and to microstructural alterations that are invisible to conventional MRI (Le Bihan, 2024).

### Diffusion functional MRI

DfMRI emerged in the early 2000s from the possibility that neuromechanical coupling could be investigated noninvasively in humans through changes in neural tissue microstructure (Darquie et al., 2001; Le Bihan et al., 2006). The dominant approach for function MRI, allowing to obtain images of brain activity, is the BOLD (Blood Oxygen Level Dependent) method (Ogawa et al., 1990, 1992), monitoring signal variations resulting from changes in blood oxygenation accompanying neuronal activity through the neurovascular coupling mechanisms (Roy and Sherrington, 1890). BOLD fMRI has major strengths, notably its noninvasiveness and ease of implementation, and it has been used extensively in cognitive neuroscience (Bandettini, 2012). However, it also has important limitations: the signal is delayed, shaped by vascular anatomy, and not strictly local to the underlying cellular event (Logothetis et al., 2001; Sirotin and Das, 2009). More fundamentally, the specificity of the BOLD signal as a proxy for neural activity has been questioned (Logothetis et al., 2001; Epp et al., 2025). DfMRI, by contrast, asks whether activation changes tissue geometry rapidly and consistently enough to alter water mobility at the cellular and tissue scales. This idea is rooted in neuromorphological, or neuromechanical, coupling, according to which neural activity is accompanied by subtle but measurable deformations of neurons, dendrites, spines, astrocytes, and extracellular space (Le Bihan et al., 2006; Stroman et al., 2008; Tsurugizawa et al., 2013; Nunes et al., 2021).

A substantial body of preclinical work has strengthened the biological plausibility of a direct tissue component in DfMRI. In hippocampal slice experiments, diffusion-sensitive signals changed with neuronal activation even in the absence of vasculature (Flint et al., 2009), indicating that microstructural tissue events alone can generate MRI-visible contrast. In rat cortex, diffusion changes closely tracked neuronal activity and persisted despite pharmacological disruption of neurovascular coupling (Tsurugizawa et al., 2013). Rapid-onset DfMRI components have also been reported with time courses more consistent with local tissue deformation than with classic haemodynamic delay (Le Bihan et al., 2006; Stroman et al., 2008; Nunes et al., 2021). This biological plausibility is reinforced by non-vascular preparations in which mechanical events can be observed directly. In squid giant axons, piezoelectric and optical measurements showed that the action potential is accompanied by rapid, reversible swelling and surface displacement (Iwasa and Tasaki, 1980; Tasaki and Iwasa, 1982). Optical studies in hippocampal slices extended this view from single axons to tissue, showing that intrinsic signal changes localized to activated postsynaptic territories track cell-volume shifts and that GABAA-dependent chloride influx contributes to the CA1 volume response (Andrew and MacVicar, 1994; Takagi et al., 2002). In avascular Aplysia ganglia, single-neuron diffusion MR microscopy demonstrated activation-induced swelling of identified neurons together with increased intracellular water mobility and reduced whole-ganglion diffusion (Abe et al., 2017). Together, these findings indicate that activation can alter neural tissue geometry directly, independently of haemodynamic coupling.

The cellular contributors most likely to generate such signals are highly ramified, membrane-rich structures. Small changes in shape, membrane tension, or extracellular spacing can disproportionately affect diffusion restriction because water molecules repeatedly encounter these boundaries during the diffusion time. Dendrites and spines are especially compelling candidates in cortex and hippocampus, where excitatory processing is dominated by spine-bearing dendritic trees and where spine density in grey matter is extremely high (Nimchinsky et al., 2002). Astrocytic fine processes are also likely contributors because they envelop synapses, regulate ionic homeostasis, and express water channels linked to volume shifts (see above). Indeed, diffusion MRI has been shown to be sensitive to astrocyte activity (Debaker et al., 2020). This complexity does not weaken the concept of neuromechanical coupling; instead, it reflects the fact that neural computation is implemented by multicompartment cellular assemblies.

The earliest human studies of DfMRI suggested that neuronal activation can produce a rapid diffusion-sensitive signal component preceding the expected haemodynamic response (Darquie et al., 2001; Le Bihan et al., 2006). More recent work has further suggested that DfMRI may capture aspects of white-matter activity or connectivity that are difficult to resolve with BOLD (Spencer et al., 2025). If confirmed, this would extend the mechanobiological framework beyond synapses and gray matter to conduction pathways themselves. In AD, where white-matter disruption, myelin change, and network disconnection are increasingly recognized (Bartzokis, 2004; Nasrabady et al., 2018), altered mechanical responsiveness of axonal tracts could contribute to temporal desynchronization and reduced cognitive efficiency even before frank disconnection becomes visible on structural imaging.

DfMRI thus appears as a natural bridge between systems neuroscience and tissue mechanics. For Alzheimer research, its significance is not merely methodological. It raises the possibility of an imaging-accessible phenotype of synaptic and dendritic mechanical dysfunction that may precede overt atrophy (Terry et al., 1991; Jackson et al., 2019). If activity in vulnerable circuits normally produces coordinated nanoscale-to-microscale deformation, and AD disrupts the structures required for that deformation, then altered DfMRI responses could represent an early marker of synaptic fragility. In that sense, DfMRI could complement amyloid and tau biomarkers by reporting not the presence of pathology itself, but the integrity of the micromechanical substrate through which memory networks operate.

### Diffusion MRI biomarkers of Alzheimer-related microstructural injury

Diffusion MRI findings fit naturally within this broader mechanobiological framework because, across normal aging and AD, their sensitivity to microstructure makes it possible to detect tissue alterations before overt macroscopic atrophy, which is one reason diffusion MRI has become central to models of brain aging (Sullivan and Pfefferbaum, 2006; Gong et al., 2014). Newer models extend beyond conventional diffusion tensor imaging (DTI) toward richer descriptions of free water, neurite architecture, and non-Gaussian diffusion (Sullivan and Pfefferbaum, 2006; Schilling et al., 2022; Steven et al., 2014). They indicate that AD is associated not only with cortical dysfunction but also with early and spatially selective breakdown of white-matter microstructure (Raghavan et al., 2021). Because hippocampal and limbic networks are affected early, diffusion MRI is especially valuable for detecting network-level injury that may precede overt atrophy on conventional structural imaging. One of the characteristic features of Alzheimer’s disease is the loss of memory, due to the early involvement of the hippocampi, but other brain regions are affected as shown by DTI which also reveals abnormalities of connections. Patients with Alzheimer’s disease often present temporo-spatial disorientation disorders and are often obsessed by the time that escapes them, clinging to their watch or clock as if it were a cane to pace their daily life. While autistic people have an impoverished contact with others, Alzheimer’s patients gradually lose contact with themselves, their memories disappearing behind a horizon, like that of a black hole.

It also suggests the next scale transition in the framework from microdomain dysfunction to tissue-level mechanics. Across studies, the most consistent pattern is reduced fractional anisotropy together with increased mean diffusivity and often increased radial diffusivity in limbic and posterior association pathways, especially the fornix, parahippocampal and posterior cingulum, uncinate fasciculus, splenium of the corpus callosum and adjacent temporo-parietal white matter (Acosta-Cabronero et al., 2010; Acosta-Cabronero et al., 2012; Buono et al., 2020). These abnormalities are often detectable in amnestic mild cognitive impairment and may precede overt hippocampal atrophy, with fornix damage appearing particularly early and lower cingulum fractional anisotropy predicting subsequent conversion to Alzheimer’s disease, supporting the view that diffusion MRI can reveal tissue injury at a stage when conventional structural MRI remains relatively insensitive (Acosta-Cabronero et al., 2010; Acosta-Cabronero et al., 2012). In autosomal dominant AD, white-matter diffusion alterations have been observed before symptom onset, reinforcing the idea that microstructural disruption is an early component of the disease process rather than merely a late consequence of cortical degeneration (Araque Caballero et al., 2018). Absolute diffusivity measures may be more sensitive than fractional anisotropy alone at symptomatic onset, whereas progressive radial diffusivity increase and fractional anisotropy decline may better track disease severity over time (Chen et al., 2023; Acosta-Cabronero et al., 2010; Acosta-Cabronero et al., 2012; Buono et al., 2020; Zhuang et al., 2013; Fu et al., 2014).

More recent diffusion approaches extend classical DTI by modelling extracellular free water and gray-matter microstructure. These studies suggest that diffusion abnormalities relate not only to tissue loss but also to Alzheimer-specific biology. Free-water measures and advanced diffusion metrics have been associated with tau-predominant signal, plasma Aβ42/40 and p-tau181, cognition and longitudinal decline (Nakaya et al., 2022; DeSimone et al., 2024). Taken together, diffusion MRI provides a scalable, non-invasive readout of Alzheimer-related tissue injury that complements structural MRI and molecular PET, although its biological specificity remains limited because vascular small-vessel disease can also contribute substantially to diffusion abnormalities in memory-clinic populations (Araque Caballero et al., 2018; Nakaya et al., 2022; Wang et al., 2023; Finsterwalder et al., 2020). An especially pertinent recent development is constrained neurite orientation dispersion and density imaging (C-NODDI), which yields an axonal density index (ADI) and a physiologically realistic estimate of white-matter axonal integrity. In a longitudinal ADNI multishell diffusion MRI study spanning cognitively normal and cognitively impaired participants, whole-brain ADI derived from C-NODDI differentiated cognitively impaired from cognitively normal individuals, detected axonal degeneration prior to overt brain atrophy, predicted subsequent decline in MMSE and CDR-SB, and tracked longitudinal clinical worsening with performance that was comparable to, and in some analyses stronger than, amyloid-PET, tau-PET and CSF biomarkers. For the present framework, the interest of C-NODDI is that it moves diffusion MRI closer to a mechanistically interpretable biomarker of axonal structural failure while retaining the practical advantages of MRI: it is non-invasive, repeatable, radiation-free and more scalable than PET for longitudinal stratification and intervention studies (Gong et al., 2026).

## Brain softening as a mesoscale and macroscale phenotype

On the other hand, Magnetic resonance elastography (MRE) has shown that the brain is a viscoelastic organ whose stiffness varies across regions and changes systematically with age (Muthupillai et al., 1995). In healthy ageing, most studies find progressive brain softening with age, and this softening is associated with lower cognitive performance (Hiscox et al., 2018; Coelho and Sousa, 2022). Age effects are region-specific, with particularly strong changes in frontal and temporal gray matter, deep gray matter structures, and the hippocampus (Hiscox et al., 2018; Delgorio et al., 2021; Feng et al., 2024). In particular, medial temporal viscoelasticity correlates with relational memory performance (Schwarb et al., 2016), and hippocampal-subfield measurements reveal age-sensitive changes in compartments that are especially vulnerable during cognitive ageing (Delgorio et al., 2021; Pavuluri et al., 2025). Together, these findings support the idea that elasticity is not merely a by-product of macroscopic atrophy, but also a marker of distributed microstructural degeneration and functional reserve in memory-relevant networks (Coelho and Sousa, 2022; Feng et al., 2024).

A mechanobiological explanation of regional softening follows naturally from synaptic- and cellular-scale changes. Ageing reduces or disrupts load-bearing microstructure, including myelin, axonal integrity, dendrites, synapses and cytoskeletal organization. Neuronal shrinkage, synaptic loss, dendritic simplification and white-matter degeneration reduce tissue packing density, making the brain less like a tightly connected cellular gel and more like a partially rarefied porous medium. Demyelination, neuritic fragmentation, extracellular remodeling and altered interstitial water balance would all contribute to a softer and more dissipative material response. Regional stiffness maps would then represent a mesoscale integration of many microscale failures.

Age-related softening is also likely to reflect a shift in tissue composition and coupling. Atrophy, enlarged extracellular and perivascular spaces, and increased CSF fraction raise the fluid-to-solid ratio, reducing apparent shear stiffness and increasing mechanical compliance. In parallel, extracellular matrix components such as hyaluronan, proteoglycans and perineuronal nets are remodeled with age; this does not simply imply less matrix, because some components may accumulate locally, but the network may become less organized, less dynamically coupled to cells, and less able to transmit mechanical stress coherently. Ageing astrocytes and microglia also change their morphology, cytoskeleton, inflammatory state and vessel interactions. Altered perivascular astrocytic endfeet, aquaporin-4 polarization, blood–brain barrier integrity and perivascular spaces can weaken fluid–solid coupling within the parenchyma.

The medial temporal lobe is especially compelling because it sits at the intersection of these literatures: it is where early tau pathology emerges, where memory symptoms are anchored, and where longitudinal MRE work suggests that lower stiffness predicts future decline (Coelho and Sousa, 2022; Feng et al., 2024; Pavuluri et al., 2025). This raises the possibility that hippocampal and parahippocampal softening reflects a cumulative reduction in local circuit resilience (Schwarb et al., 2016; Delgorio et al., 2021). Such measures would not replace amyloid or tau biomarkers, but they might index the degree to which pathology has already destabilized the local tissue environment in functionally meaningful ways (Coelho and Sousa, 2022; Feng et al., 2024).

In this respect, brain mechanics could be regarded as a convergent phenotype of synaptic pathology, but also of vascular ageing and fluid imbalance (see below). Indeed, vascular factors appear to shape local brain mechanics. Large-artery stiffening, altered pulse pressure and microvascular dysfunction are increasingly recognized as modifiers of dementia risk, and they alter the physical forcing applied to perivascular spaces and the stress environment experienced by parenchyma (Feng et al., 2024; Pavuluri et al., 2025). Higher pulsatility and vascular burden have been associated with lower hippocampal stiffness, consistent with the idea that arterial ageing progressively damages the mechanical environment of memory circuits (Coelho and Sousa, 2022; Feng et al., 2024). A chronically altered pulsatile regime may progressively damage the mechanical coherence of vulnerable tissues while simultaneously compromising clearance of toxic wastes such as tau and amyloid-beta proteins. In the present framework, this is important because it provides a route by which cardiovascular health can influence cognition not only through perfusion or infarction, but also through the physical state of the tissue itself (Coelho and Sousa, 2022).

**Figure 3:**
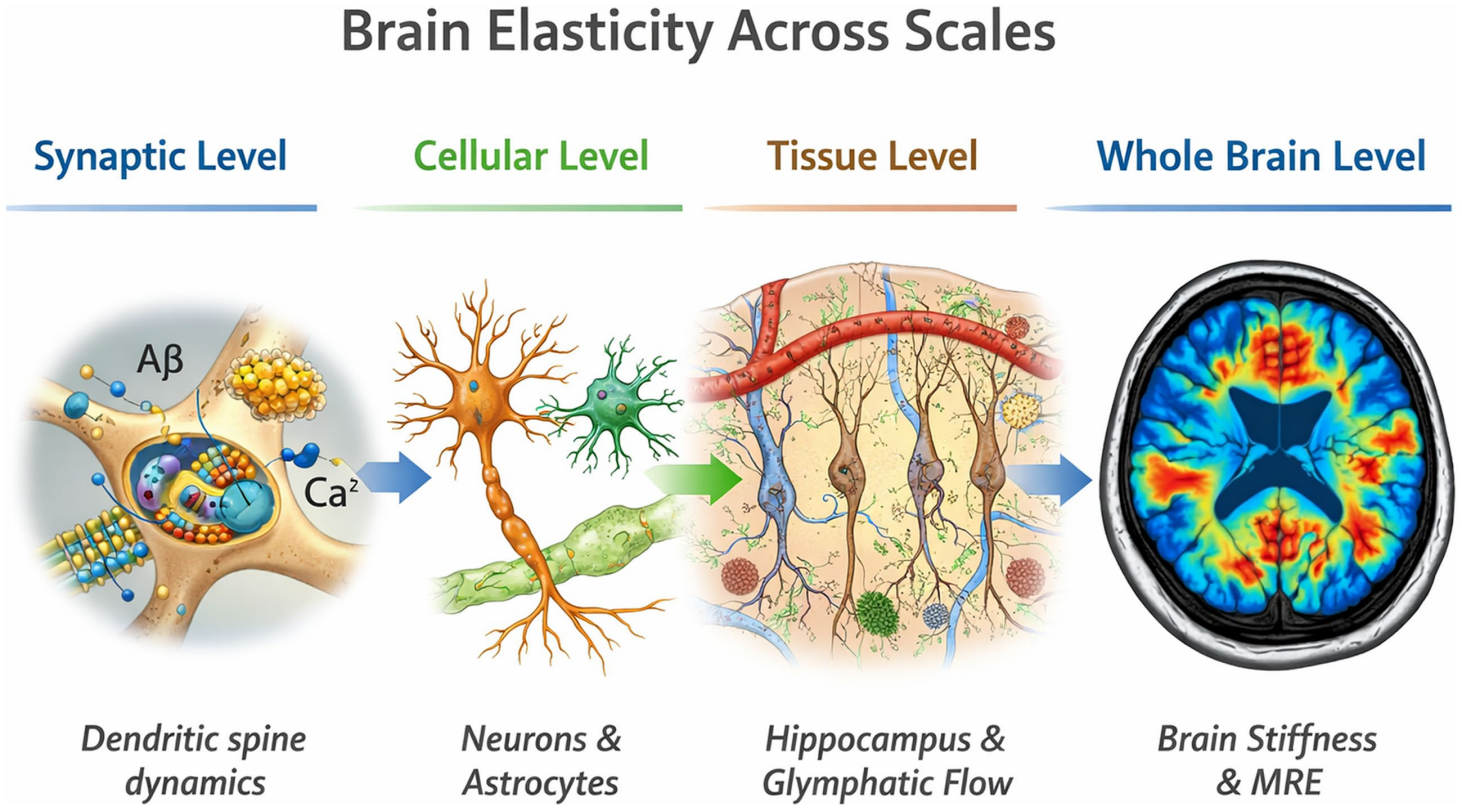
Brain elasticity across scales.

In this framework, brain mechanics provides a variable multiscale interface linking vascular ageing, synaptic vulnerability and cognitive decline. Age-related brain softening can be understood as the combined consequence of loss of solid microstructural scaffolding, reduced cellular and synaptic density, increased fluid fraction, extracellular-matrix remodeling, glial/perivascular uncoupling, vascular ageing and impaired fluid–solid coupling (Figure 3). It therefore offers a potential multiscale readout of how molecular, cellular, vascular and hydrodynamic processes converge to weaken the mechanical resilience of ageing and Alzheimer-vulnerable brain tissue (Coelho and Sousa, 2022; Feng et al., 2024; Pavuluri et al., 2025).

An issue with conventional MRE, however, is that it relies on externally induced mechanical waves whose propagation is monitored with MRI to estimate shear stiffness (Muthupillai et al., 1995). Such vibrations might not be suitable in already compromised brains. Weaver and colleagues showed that intrinsic brain motion induced by cardiac pulsations can serve as an effective source for mechanical property estimation, suggesting that brain stiffness might be measured without dedicated external actuation (Weaver et al., 2012). Zorgani et al. later extended this concept with a passive, broadband ‘brain palpation’ approach that extracted elastic information from naturally occurring physiological vibrations, effectively adapting seismic-noise and time-reversal concepts to *in vivo* brain MRI (Zorgani et al., 2015). However, these studies were primarily methodological. More generally, MRE, whether based on external or intrinsic vibrations, faces practical limitations related to skull attenuation, reflection and diffraction, limited spatial coverage, and wavelength-constrained spatial resolution (Low et al., 2016).

An alternative is diffusion-based virtual MRE, which infers tissue stiffness from diffusion MRI without requiring externally induced shear-wave propagation (Le Bihan et al., 2017). The conceptual basis of vMRE is that the same microstructural features that hinder water diffusion in tissues, such as cellular density, axonal packing, myelination, extracellular matrix organization and interstitial geometry, also contribute to tissue mechanical stiffness (Le Bihan et al., 2017). Diffusion-based vMRE therefore uses optimized diffusion measurements, particularly a shifted apparent diffusion coefficient (sADC), which is more sensitive to tissue microstructure and is linearly and negatively correlated with shear stiffness, allowing conversion into stiffness values expressed in kPa (Le Bihan et al., 2017). This approach can provide accurate, high-resolution stiffness maps without external mechanical vibration and was first validated in the liver for fibrosis staging (Le Bihan et al., 2017; Kromrey et al., 2020). In brain imaging, it may provide clinically relevant information in neurosurgery, aging and neurodegeneration. Early studies suggest that vMRE-derived stiffness can predict tumor consistency in pituitary adenomas and meningiomas, which could improve preoperative planning and surgical risk assessment (Lagerstrand et al., 2021; Aunan-Diop et al., 2023; He et al., 2025). IVIM-based vMRE further expands this concept by simulating virtual mechanical waves from stiffness maps, thereby creating a programmable, elasticity-driven contrast that could reveal pathological heterogeneity not visible with conventional imaging (Le Bihan et al., 2017).

Interestingly, functional MR elastography (fMRE) is an emerging extension of brain MRE designed to detect transient mechanical changes associated with neuronal activation. The central observation is that cortical stiffness can change during sensory or motor stimulation, suggesting that viscoelastic properties are modulated by physiological activation. In the first simultaneous human fMRI-fMRE visual study, stiffness within the visual cortex increased by approximately 6–11% during checkerboard stimulation (Lan et al., 2020). This study established that fMRE can map activation-related stiffening in human cortex (Palnitkar et al., 2024) and that the effect is aligned with the decrease in ADC and sADC observed with diffusion fMRI (Le Bihan et al., 2006), given the linear negative correlation between ADC or sADC and shear stiffness. Preclinical work by Patz et al., 2019 further suggests that stimulus-locked biomechanical changes occur on much faster timescales than the neurovascular-coupling-driven response measured with BOLD fMRI. This timing is also consistent with the fast, neural-locked response reported with diffusion fMRI (Le Bihan et al., 2006; Tsurugizawa et al., 2013; Nunes et al., 2021).

In that context, diffusion-based virtual MRE appears as an attractive, scalable non-invasive biomarker to investigate brain stiffness in the ageing brain and for longitudinal or interventional studies of AD, including intervention stratification. The method could be especially useful if early-stage trials begin to combine molecular endpoints with markers of tissue compliance and transport, but would require further calibration (rescaling), especially because the diffusion–stiffness calibration has been established mainly in the liver (Le Bihan et al., 2017) and brain tumors (He et al., 2025). Furthermore, due to the anisotropic nature of brain tissue, especially in white matter, diffusion-based virtual MRE needs to rely on DTI acquisitions, so as to estimate shear-stiffness from radial sADC (obtained perpendicularly to fiber directions).

## Glymphatic transport and tissue compliance

The glymphatic system extends the mechanistic framework further into fluid transport. This multicomponent system is a brain-wide perivascular transport and clearance pathway in which cerebrospinal fluid enters along periarterial spaces, exchanges with interstitial fluid within the parenchyma, and exits along perivenous and meningeal lymphatic routes. This process depends on arterial pulsatility, CSF dynamics, perivascular space, astrocytes and aquaporins (AQP4) activity. In humans, MRI is currently the main investigative platform, using intrathecal or intravenous tracer approaches as well as non-contrast surrogates such as diffusion tensor imaging along the perivascular space (DTI-ALPS), although these methods remain indirect and not fully standardized (Naganawa et al., 2024; Boyd et al., 2024). Interestingly, using those approaches, it has been shown that psychiatric disorders may involve impaired brain clearance and neuro-glio-vascular homeostasis, not only altered neurotransmission (Barlattani et al., 2025).

The link with Alzheimer’s disease (AD) is biologically plausible because glymphatic and meningeal lymphatic dysfunction may reduce clearance of amyloid-β, tau, and inflammatory mediators, while ageing, sleep fragmentation, vascular stiffening, and loss of aquaporin-4 polarization can further impair fluid exchange and perivascular transport (Ding et al., 2025). Human studies increasingly support this association. MRI-based glymphatic measures have been linked to AD-related changes in cognition and cerebrospinal fluid proteomic signatures in amnestic mild cognitive impairment, suggesting that impaired clearance may be detectable before established dementia (Cullell et al., 2025). In addition, a randomized crossover human sleep study reported that normal sleep increased morning plasma amyloid-β and tau relative to sleep deprivation, consistent with enhanced overnight glymphatic clearance and providing a mechanistic bridge between sleep disruption and AD risk (Dagum et al., 2026). Overall, current evidence supports the view that glymphatic dysfunction is unlikely to be the sole cause of AD, but may act as a disease-modifying mechanism that interacts with amyloid, tau, vascular ageing, and neuroinflammation, making it a promising target for biomarker development and early-stage intervention (Ding et al., 2025; Boyd et al., 2024).

The key mechanobiological point, here, is that glymphatic transport is not purely a fluid phenomenon. It depends on how pulsatile forces are transmitted through the tissue and how compliant the surrounding parenchyma and perivascular walls are. Brain softening, vascular stiffening and altered extracellular architecture could therefore change the efficiency or distribution of clearance. Conversely, impaired clearance could feed back on tissue organization through protein deposition, astrocytic dysfunction and inflammatory remodeling. The result is a coupled fluid–solid system in which tissue mechanics and waste transport are mutually dependent. This interpretation also helps explain why sleep is so central. Sleep disruption is common in early AD and is increasingly viewed as both a symptom and a contributor to disease progression. In a purely molecular framework, poor sleep is often discussed because it increases neuronal activity or changes soluble Aβ production. In a mechanobiological framework, sleep also matters because it changes glymphatic state, extracellular space behavior and perhaps the mechanical operating regime of the tissue. That is, the sleeping brain may not merely produce less pathological stress; it may physically clear and recover differently (Ding et al., 2025).

**Figure 4:**
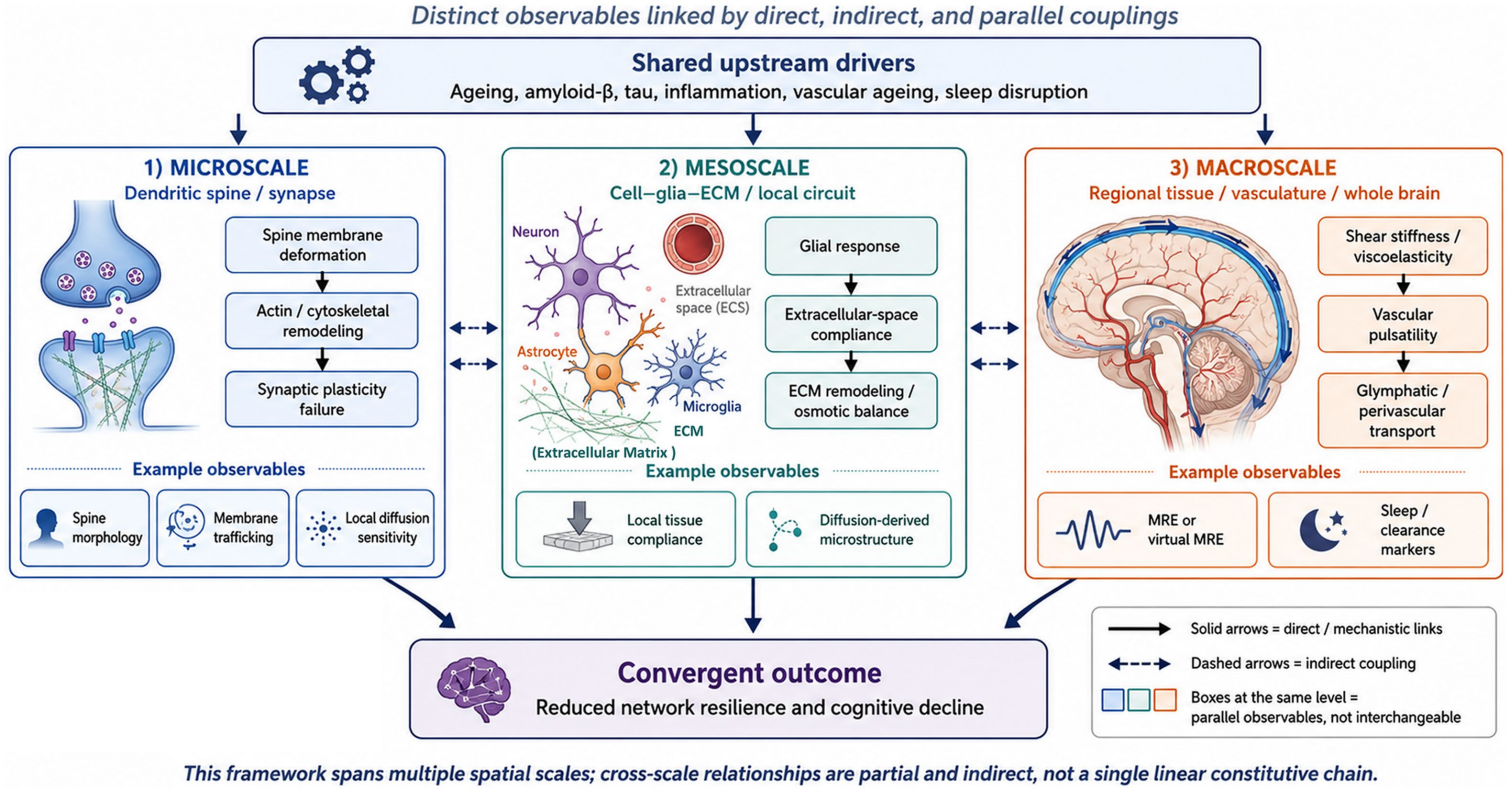
Overall multiscale mechanobiological framework in Alzheimer’s disease.

At the modelling level, one long-term goal would be to connect measurements across scales (Figure 4). Spine dynamics studied optically, activity-dependent diffusion changes measured with DfMRI, regional viscoelasticity measured with MRE or vMRE, and clearance dynamics inferred from glymphatic imaging are often investigated in separate literatures. The mechanobiological framework predicts that they should not be independent. A future quantitative model might describe how nanoscale morphological compliance aggregates into mesoscale diffusional responses and finally into regional material properties, with vascular pulsatility and sleep state acting as modulators of the entire system (O’Donnell et al., 2011; Araya et al., 2014; Iliff et al., 2012; Iliff et al., 2013; Ding et al., 2025; Naganawa et al., 2024).

## Testable mechanistic intervention: forty-hertz stimulation

**Figure 5:**
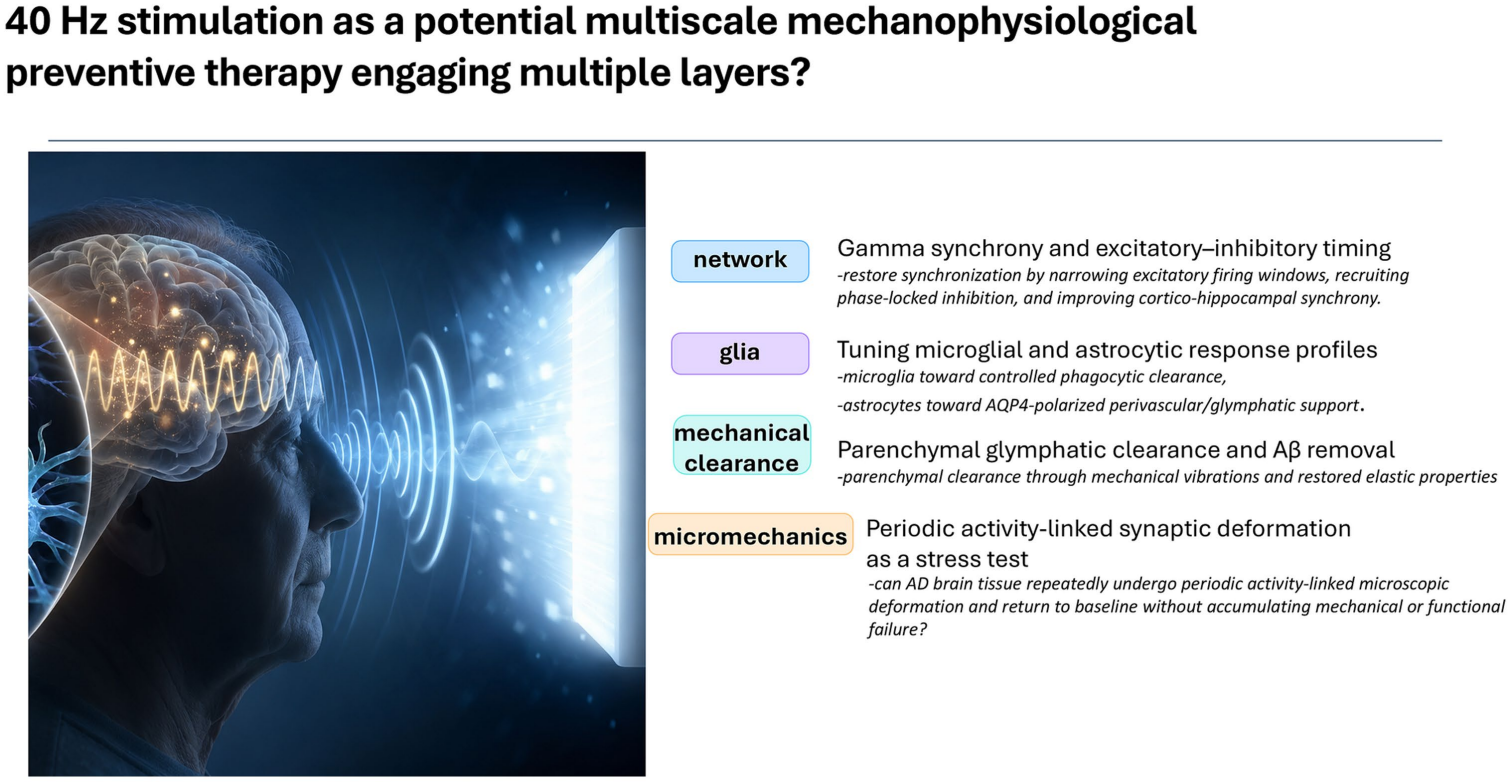
Preclinical studies have suggested that 40 Hz sensory stimulation could be used as an early-stage AD intervention candidate. Hypothetical mechanisms have been proposed, which could work in combination.

The emergence of 40 Hz stimulation in AD research is especially interesting in this context. Sensory and electrical 40 Hz stimulation paradigms were originally motivated by abnormalities of gamma-band synchrony in AD and by preclinical evidence that gamma entrainment can influence amyloid burden, glial behavior and circuit function. Forty-hertz (40-Hz) gamma oscillations are generated by synchronized neural network activity and appear attenuated in Alzheimer’s disease patients and transgenic mouse models (Palop et al., 2007). Interestingly, diffusion fMRI signals might be sensitive to such gamma oscillations (Tsurugizawa et al., 2017). In transgenic AD mice, 40-Hz visual stimulation reduced amyloid burden in visual cortex (Iaccarino et al., 2016), although effects of visual stimulation have remained controversial (Soula et al., 2023). Subsequent multisensory stimulation extended these effects to the temporal cortex and hippocampus while improving cognition (Martorell et al., 2019). Related studies from other groups likewise reported neuroprotective or cognitive benefits of 40-Hz stimulation (Adaikkan et al., 2019; Etter et al., 2019; Park et al., 2020). More recently, multisensory gamma stimulation was considered to enhance aquaporin-4 polarization at astrocytic endfeet, supporting directional water exchange at the gliovascular interface, arterial pulsatility promoting interstitial solute washout (VIP interneurons are implicated as one route linking gamma entrainment to vascular pulsatility in mice AD models) and meningeal lymphatic downstream drainage, thereby promoting glymphatic clearance of amyloid. Multisensory 40 Hz stimulation was found to increase CSF influx into cortex and interstitial-fluid efflux, consistent with recruitment of glymphatic-like clearance pathways. Blocking glymphatic clearance or interfering with AQP4 function attenuated the amyloid-removal effect (Murdock et al., 2024). Thus, 40 Hz stimulation is already situated at the interface of network dynamics, glial biology and fluid handling (Iaccarino et al., 2016; Martorell et al., 2019; Murdock et al., 2024; Sun et al., 2024; Cantoni et al., 2025; Park and Tsai, 2025). Within the mechanobiological framework proposed here, 40 Hz stimulation can be viewed more broadly as an intervention that may restore oscillatory coordination across electrical, glial, vascular and mechanical domains. At the neuronal level, gamma entrainment may help normalize fast inhibitory–excitatory timing and thereby stabilize activity-dependent micromechanical responses in dendrites and spines. At the glial and perivascular level, it may influence astrocytic responses, vascular pulsation patterns or clearance pathways (Figure 5). Importantly, those mechanisms are supported mainly by animal-model data; whether the same magnitude and pathway operate in humans with AD yet remains unresolved.

Still, one may envision another hypothetical mechanism directly linked to the viscoelastic nature of the brain parenchyma at the whole-brain scale. This mechanistic link should not be overstated, but it is physically plausible enough to justify targeted testing. At cardiac frequency near 1 Hz, whole-brain shear modulus has been estimated to be approximately 42 ± 13 Pa, consistent with a highly compliant viscoelastic parenchyma through which pulsatile forcing from blood vessels and CSF spaces can propagate over brain-scale distances during a heartbeat (Herthum et al., 2021). In that setting, age-related softening could shorten shear wavelength and weaken propagation dynamics that are relevant to glymphatic transport. Because in viscoelastic systems stiffness physically increases with vibration frequency, one may consider whether the induction of soft mechanical vibrations to the brain parenchyma near the gamma range, around 40 Hz, through the skull bone, for instance using common bone-conduction headsets positioned at the temporo-mandibular junction, might also partially restore effective tissue mechanics and fluid–solid coupling and, hence, glymphatic system efficiency. Indeed, Jindrak and Jindrak proposed decades ago that skull vibrations generated by loud vocalization might facilitate CSF-mediated clearance [Jindrak and Jindrak, 1988]. Of historical interest, “vibratory therapeutics” was originally proposed by Charcot at the end of the 19st century for the treatment of some neurological disorders, notably Parkinson’s disease (Charcot, 2011).

These lines of evidence do not prove that therapeutic gamma stimulation works through a mechanical route in AD and bone-conducted stimulation is advanced only as a mechanistic hypothesis, which will require future experimental tests, not as a clinical claim. However, they make the fluid–solid coupling hypothesis reasonable enough to test directly. Junowicz et al. showed that mechanical vibration can alter protein separation in gel-filtration systems, a simplified but useful physical analogy (Junowicz et al., 1972). The hypothesis is further motivated by work in aged rhesus monkeys showing that one week of daily 40-Hz auditory stimulation increased CSF amyloid-beta levels for weeks after treatment, consistent with mobilization from brain parenchyma, although the viscoelastic interpretation was not the focus of that study (Wang et al., 2026). This does not imply that 40-Hz stimulation is an established treatment for AD. Human studies remain early-stage, effects are modest, and mechanistic specificity is unresolved. Nevertheless, the intervention is conceptually important because it is one of the first plausible therapies that can be interpreted simultaneously in electrophysiological, glial, glymphatic, and mechanical terms.

The same logic argues for combination therapy. Anti-amyloid agents may reduce biochemical load, while sleep optimization, vascular protection and perhaps gamma-based stimulation preserve the physical and physiological substrate through which memory circuits function. If AD is indeed partly a failure of coupled mechanical resilience, then therapies aimed only at protein removal may be insufficient once the tissue has already lost its adaptive mechanical capacity. Indeed, a recent review of 17 studies, involving 20,342 volunteers who received recent drugs (specifically Donanemab and Lecanemab), shows that the successful removal of amyloid from the brain does not seem to be associated with clinically meaningful effects in people with mild cognitive impairment or mild dementia due to AD (Nonino et al., 2026). This is one reason a mechanobiological perspective may be most valuable for prevention and prodromal disease rather than late dementia.

## Discussion: translational implications, caveats, and testable predictions

**Figure 6:**
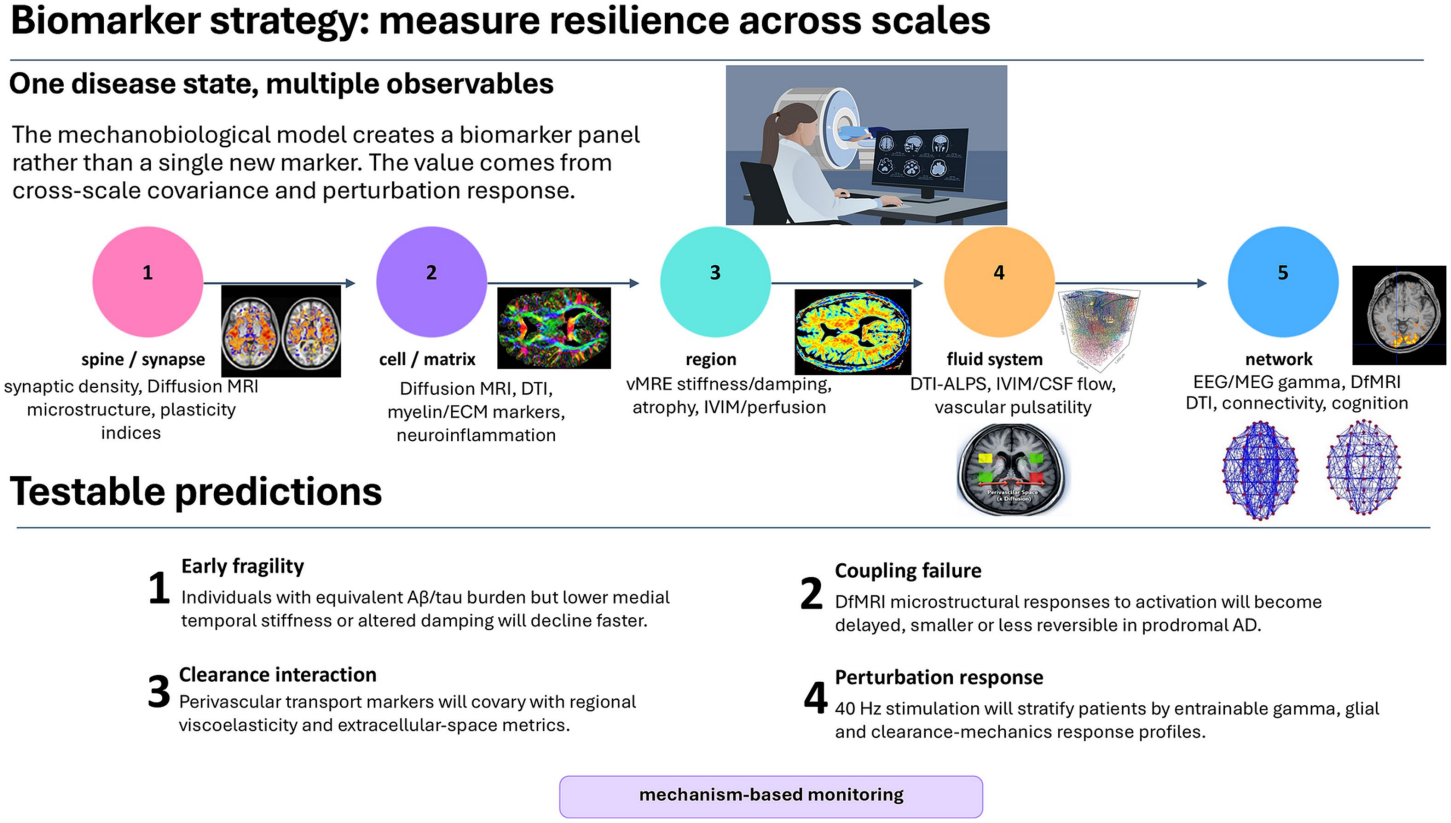
Link between measurable observables across brain scales and corresponding imaging biomarkers, and main testable predictions supported by the mechanobiological framework.

A useful scientific framework should do more than connect ideas retrospectively; it should generate specific predictions. The present model does so. First, early AD and biomarker-positive mild cognitive impairment should show measurable alterations in medial temporal mechanics that correlate with cognition and synaptic biomarkers more closely than amyloid burden alone. Second, diffusion-based functional measures of neuromorphological coupling should be abnormal in vulnerable memory networks before major atrophy emerges. Third, glymphatic impairment should covary with both sleep disruption and regional softening, especially in structures exposed to strong vascular ageing effects. Fourth, interventions that improve gamma synchrony should also influence markers of fluid transport and perhaps tissue mechanics, not only electrophysiological readouts (Figure 6).

These predictions immediately suggest a research agenda. Longitudinal cohorts should combine amyloid and tau biomarkers with MRE or virtual MRE, sleep and glymphatic imaging markers, and diffusion-based functional or microstructural measures. Early-stage interventional studies should test whether treatments that improve sleep, vascular function or gamma entrainment stabilize regional stiffness or preserve diffusion-derived markers of local tissue responsiveness. Animal models should directly relate spine-level structural plasticity, local tissue mechanics and clearance under controlled amyloid/tau conditions. Such studies would not merely refine the mechanobiological hypothesis; they would determine whether it deserves a central place in the next generation of AD theory.

The framework also has a pragmatic clinical implication: biomechanics-sensitive measures may become useful as integrative biomarkers. Amyloid and tau biomarkers indicate molecular pathology, but they do not directly report the resilience of the tissue that must continue to function in the presence of that pathology. Measures of stiffness, viscoelasticity and neuromorphological response in a noninvasive manner through diffusion MRI may capture a different and potentially more actionable dimension: whether the network still retains enough physical integrity to respond to treatment or compensation. In that sense, the mechanobiological framework is not only explanatory; it is a proposal for broadening what counts as meaningful disease staging in AD. The key conceptual terms of the proposed mechanobiological framework are summarized in Table 1, with links of each conceptual construct to its biological interpretation, candidate measurement modality, and expected disease-associated changes.

**Table 1:** Key conceptual terms of the proposed mechanobiological framework.

Conceptual term	Definition	Biological interpretation	Candidate measurement modality	Expected change in AD
Mechanical resilience	Capacity of brain tissue to deform, recover, and preserve function under stress at multiple scales.	Ability to maintain spine geometry, tissue integrity, and perivascular transport despite inflammatory, or molecular stress.	MRE or vMRE; diffusion-fMRI; longitudinal structural MRI; cognitive-stress paradigms.	Reduced recovery, greater deformation vulnerability, regional softening, especially in memory networks.
Mechanical reserve	Latent biomechanical buffer before structural or functional failure occurs.	Mechanical analogue of cognitive reserve: tissue can tolerate pathology while preserving synaptic and network function.	Longitudinal MRE or vMRE; cognition, hippocampal volumetry.	Reserve depletion; same molecular load produces greater cognitive impact when stiffness/resilience is low.
Synaptic mechanocompetence	Ability of synapses to undergo dynamic mechanical remodeling to support function.	Actin remodeling, membrane tension, spine head/neck geometry, receptor trafficking, and postsynaptic density organization remain coordinated.	Diffusion-fMRI	Spine instability, impaired LTP/LTD, reduced receptor organization, early synaptic failure before atrophy.
Mechanical operating range	Range of stiffness/compliance within which tissue mechanics remain adaptive.	Brain tissue must be neither excessively rigid nor excessively compliant; normal signaling requires reversible deformation and recovery.	Regional MRE or vMRE stiffness/viscosity maps; diffusion metrics of microstructural compliance.	Shift toward excessive compliance/softening, increased dissipation, delayed recovery after activation.
Fluid–solid coupling	Interaction between cellular/ExtraCellular Matrix mechanics and interstitial/perivascular fluid movement.	Parenchymal stiffness, vascular pulsatility, astrocytic endfeet, AQP4 polarization, extracellular space, and CSF–ISF exchange act together.	glymphatic MRI; diffusion MRI; MRE or vMRE; AQP4/perivascular markers in models.	Impaired glymphatic/perivascular transport, enlarged perivascular spaces, altered pulsatility, reduced Aβ/tau clearance.

There are also immediate implications for animal models. Much preclinical AD work quantifies plaques, tangles, synapse number or behavior, but relatively little measures material properties of tissue, local swelling responses during activation or the interaction between pathology and mechanical loading. Incorporating such measures could improve translation by revealing whether candidate therapies preserve the physical adaptability of tissue in addition to changing molecular endpoints. This may be particularly important for interventions expected to work early, when functional rescue is still plausible despite modest histological change.

Finally, the framework suggests that therapeutic development should move beyond a single-endpoint logic. If a treatment reduces amyloid modestly, improves sleep, stabilizes regional stiffness and enhances glymphatic transport, those effects may be synergistic even if none is dramatic in isolation. Conversely, a therapy that lowers protein burden but leaves tissue mechanically fragile may underperform clinically. Future early-stage trials could therefore benefit from composite endpoint strategies that combine molecular biomarkers with measures of tissue resilience, network function and fluid transport, as possible with diffusion MRI.

A major caveat remains that current evidence is not yet sufficient to claim that altered neuronal or synaptic mechanics are primary causes of AD. The model is best regarded as a structured hypothesis that organizes disparate findings into a coherent systems account. Its value lies precisely in the fact that it is falsifiable. If future work shows that regional mechanics add no information beyond atrophy, that diffusion fMRI abnormalities do not relate to synaptic markers, or that glymphatic alterations are independent of tissue compliance, then the framework will need revision. But if the predicted couplings are confirmed, AD research may gain an entirely new layer of mechanism and therapeutic logic.

Ultimately, the attractiveness of a mechanobiological framework lies in its ability to connect mechanical features occurring at different scales, from synaptic physiology to glymphatic clearance (Figure 4). The relationships among those different scales and mechanisms should be understood as a set of direct, indirect, and parallel couplings rather than as a single constitutive chain. At the microscale, amyloid-β and tau may alter cytoskeletal organization, membrane trafficking, and dendritic-spine geometry, thereby affecting synaptic plasticity. At the mesoscale, glial activation, extracellular-matrix remodeling, altered extracellular volume, and impaired ionic/osmotic regulation may modify local tissue compliance. At the macroscale, demyelination, vascular ageing, fluid-fraction changes, atrophy, and impaired perivascular transport may influence regional viscoelasticity and glymphatic efficiency. These levels may interact over disease progression, but each has partly distinct material determinants and measurement readouts. For instance, regional softening should not be considered as a direct readout of failed synaptic mechanics.

Thus, the proposed framework does not claim that one mechanical observable explains all others; rather, it proposes that AD involves multiple mechanically sensitive compartments whose failure modes may converge on reduced network resilience and cognitive decline. Together, they suggest that what fails in Alzheimer’s disease is not merely a set of molecules or even a set of neurons, but an integrated material system that must remain excitable, deformable, perfusable and self-clearing in order to support memory over decades. In summary, Alzheimer’s disease can be viewed not only as a proteinopathy and network disorder, but also as a progressive failure of mechanical resilience across scales. Synaptic micromechanics, neuronal and glial deformation, extracellular architecture, vascular pulsatility, glymphatic transport, and regional brain stiffness all appear capable of interacting with the more familiar molecular hallmarks of the disease. This perspective does not replace amyloid and tau biology; it embeds them in a broader multiscale systems model that may be especially relevant to early disease, when synaptic and tissue mechanics may still be modifiable.

More generally, while this proposed mechanobiological framework was anchored to the defining biological, anatomical and temporal features of Alzheimer’s disease, it would be worth considering whether and how this framework could be tuned in the future to fit other neurodegenerative disorders in which disease-specific molecular stresses could progressively impair the mechanical resilience of vulnerable neural circuits. For instance, Parkinson’s disease may involve analogous, but topographically and molecularly distinct, mechanobiological processes, centered on nigrostriatal/basal ganglia motor networks and *α*-synuclein aggregation, membrane interaction, and propagation (Henderson et al., 2019), rather than on the amyloid–tau cascades characteristic of AD. It would be, indeed, of great interest to investigate in the future whether and how the proposed framework could be tuned to fit those other neurodegenerative disorders.

In conclusion, the mechanobiological framework proposed here is therefore both conservative and ambitious. It is conservative because it incorporates established molecular and imaging findings rather than rejecting them. It is ambitious because it suggests that brain tissue mechanics may provide a missing level of integration between protein aggregation, synaptic dysfunction, clearance failure, and clinical decline. If that view proves correct, future AD therapy may need to protect not only against toxic proteins but also against the progressive loss of the tissue’s ability to deform, clear, synchronize, and recover. That possibility alone justifies treating mechanobiology not as a peripheral curiosity, but as a serious candidate framework for the next phase of Alzheimer research.

## Data Availability

The original contributions presented in the study are included in the article/supplementary material, further inquiries can be directed to the corresponding author/s.
